# Did We Get Pasteur, Warburg, and Crabtree on a Right Note?

**DOI:** 10.3389/fonc.2013.00186

**Published:** 2013-07-15

**Authors:** Lakshmipathi Vadlakonda, Abhinandita Dash, Mukesh Pasupuleti, Kotha Anil Kumar, Pallu Reddanna

**Affiliations:** ^1^Dr. CR Rao Advanced Institute of Mathematics Statistics and Computer Science, University of HyderabadHyderabad, India; ^2^SRM Research Institute, SRM University, Kattankulathur, India; ^3^Department of Animal Sciences, School of Life Sciences, University of Hyderabad, Hyderabad, India; ^4^National Institute of Animal Biotechnology (NIAB), C.R. Rao AIMSCS, University of Hyderabad Campus, Hyderabad, India

Antoine Lavoisier (eighteenth century) demonstrated that living organisms consume oxygen to slowly burn the fuels in their bodies to release energy (1). Louis Pasteur (2) in an epoch making discovery, recognized as “*the Pasteur effect*,” declared “fermentation is an alternate form of life and that fermentation is suppressed by respiration” [reviewed in (3–5)]. Yet another observation of Pasteur, that yeast cells consume oxygen to multiply, is debated less among cancer biologists. This nevertheless, forms the first scientific observation that proliferating cells need oxygen to divide. Pasteur was involved in a path breaking debate with Liebig on whether fermentation is a chemical or a biological process? By 1880s Pasteur could establish that fermentation is a physiological process, and oxygen, considered to be a putrefying factor by Liebig and others, was a growth promoting factor [reviewed in (3)]. Six decades later, Warburg (6) proposed that damaged respiration and enhanced fermentation of glucose is the prime cause of cancer formation. It became popular as “*aerobic glycolysis*” and a counter to “*the Pasteur effect*.” Warburg was uncompromising and was intolerant to any alternate theory on cancer formation and declared to the German Central Committee for Cancer control in 1955 at Stuttgart “… there is today no other explanation for the origin of cancer cells, either special or general. From this point of view, mutation and carcinogenic agent are not alternatives, but empty words, unless metabolically specified…” (7). Crabtree (8), a contemporary of Warburg, suggested that pathological over growths use aerobic glycolysis as a source of energy and glucose uptake and glycolytic activity has a depressive effect on oxygen consumption. His conclusion gained the popularity as the “inverted Pasteur effect” or the “Crabtree effect” (9). Several authors in the middle of twentieth century reported that glucose is a negative regulator of respiration. These reports indicate that there is an initial stimulation of oxygen consumption for about 20–120 s following glucose consumption followed by an inhibitory period, which after equilibration stabilizes to a constant of about 30% of the endogenous rate until all the glucose was consumed [reviewed by (9)].

## Pasteur, Warburg, and Crabtree – the Three Edges of a Triangle Coin?

A critical analysis of the statements suggests that Pasteur, Warburg, and Crabtree represent the three edges of a *Triangle Coin*, while glucose and oxygen form the two sides of the coin. Semantics apart, the three authors conclude that glucose utilization and the presence of oxygen are required for proliferating cells. Pasteur observed that when sufficient oxygen is available, yeast “seizes to be ferment and increases in mass,” but renews its capacity to ferment under depleted oxygen. His observations that ammonia transformed into a complex “*albuminoid*” (protein) compound during fermentation and growth is a recognition of the fact that the two processes can coexist when nitrogen source and oxygen are available [see the reviews (3, 4)]. Warburg's hypothesis that “*damaged respiration promotes fermentation even in the presence of oxygen*” is a reiteration of Pasteur's statement on growth in principle. Warburg considered fermentation but not oxygen is a deciding factor in proliferation. He was aware that glucose consumption and the oxygen levels in different regions of the tumors can vary and fermentation decreases in the direction of capillary blood flow (10). Tumors grafted in low oxygen tension were shown to grow slowly (11); Crabtree demonstrated 50% higher respiration in subcutaneous tumors than those in abdomen which has 50% higher oxygen tension (8). It is noteworthy that Harvey (12), around Warburg's times, demonstrated that oxygen is needed for the proper division of Sea Urchin eggs, and lack of oxygen arrests the development. Recent studies indicate that proliferative cells exhibit altered metabolism (13–15) and tumor is a heterogeneous tissue; a metabolic symbiosis exists between cells in sharing glucose and lactate between well oxygenated and low oxygenated population of cells (16).

In short, with regard to proliferative cells, there are two unifying principles in the three apparently diverging hypotheses of Pasteur, Warburg, and Crabtree, i.e., an inverse relation exists between glucose uptake and oxygen utilization (respiration); while fermenting cells require more glucose, the proliferative cells require both glucose and oxygen. Pasteur, in addition, points to the requirement of nitrogen as an additional source (albuminoid) for growth of yeast

## Adenylate Phosphate Esters (ATP, ADP, and AMPs) Regulate Metabolism

The debate on Warburg and Pasteur, gave a lead to deciphering the details of glycolysis, citric acid cycle, and the role of adenylate phosphate esters (ATP, ADP, and AMPs) in metabolic regulation [see (17–21)]. The discovery of ATP by Fiske and Subbarow and Lohmann paved the way for understanding the role of oxygen in ATP generation through mitochondrial respiration and oxidative phosphorylation (OXPHOS) [reviewed by (5)]. The debate on the mitochondrial function, reactive oxygen species (ROS), and regulation of OXPHOS, however, is still alive (22, 23). Parallel to the research on cancer cells were the studies on energetics of muscle contraction, mainly by Lundsgard, Meyerhof, A. V. Hill, and others, where lactate production, heat generation, and oxidative recovery of the energy remained the main focus [reviewed in (4)]. Some of these early researchers came out with the theories of competitive limitation of inorganic phosphates (Pi), ADP, and hexokinase as regulating agents of OXPHOS and glycolysis. Two key inhibitors, the indoleacetic acid (IAA) and nitrophenols which block glycolysis, and OXPHOS respectively, were developed to examine the relative influence of glycolysis and OXPHOS on metabolism of cells and exercising muscles which produced enormous amounts of lactate [reviewed in (4) and (24)]. Most of these researchers were influenced by either Warburg or Pasteur hypotheses. For example, Nigam (25), demonstrated that in Novikoff Ascites-Hepatoma cells, when OXPHOS is blocked in the presence of oxygen by nitrophenols, glucose consumption is rapid but reaches a plateau within 10–15 min, and glycolysis (lactate production) is up regulated at the expense of the glycogen synthesis. In the absence of nitrophenols glucose up take was proportional to the glycogen synthesis and increased slowly but exponentially when compared to the cells with blocked OXPHOS. The author tried to explain the results as a different type of Pasteur effect, but a critical analysis of the results indicates that glucose uptake in the presence of oxygen is proportional to its utility in macromolecular biosynthesis.

## The Ratio of AMP and ATP (Energy Charge) Regulates Glycolysis and Oxphos

Phosphofructokinase (PFK) was identified as a key regulator of glycolysis (4, 26). Ramaiah et al. (27) demonstrated that the ratio of AMP and ATP in cells decides whether PFK is inhibited or activated. Atkinson (28) suggested that feed back cycles regulate the metabolic fate of cells and this regulation depends on the energy charge of cells, i.e., [(ATP) + 0.5(ADP)]/[(ATP) + (ADP) + (AMP)]. The ratio of ATP production between glycolysis and mitochondrial OXPHOS is 1:15 per one molecule of glucose and it takes only few molecules of glucose for healthy cells to reach the saturating levels of ADP:ATP ratio of 1:10 (29). Mitochondria have several other functions, in addition to ATP producing function. During biosynthetic processes they act as regulators of the metabolic homeostasis in cells and regulate the carbon and nitrogen fluxes between proteins, lipids by activating anaplerotic and cataplerotic reactions for reconstruction of membranes, and supra molecular structures [see (30–33)].

## Energy Status of Cells also Modulates Cell Signaling Pathways

The explosion of genomic research that followed the discovery of DNA double helix in the last half of twentieth Century has shifted focus of cancer research from metabolism to gene mutations (14, 34–36). One of the key contributions of the genomic era is the discovery of the oncogenes and tumor suppressors (37), which work in a regulated series of networks of signaling pathways that respond to environmental cues in modulating the energy metabolism and cell cycle progression. There was a renewed search for pathways responsible for metabolic reprograming in cancer cells [see (38–40)] and Akt was named as “Warburg enzyme” (41). Akt and its downstream target, the mechanistic target of rapamycin (mTOR; earlier known as mammalian target of rapamycin), were recognized to play crucial role in several metabolic disorders including cancer [Reviewed by (42)]. The mechanistic target of rapamycin, especially the complex1 (mTORC1) plays a crucial role in regulation of metabolism and promotes the biosynthetic activity of cells (an energy consuming process) and activates cell survival pathways by inhibiting GSK3β and autophagy. It has come to be recognized that ATP/ADP ratio plays critical role in modulating the functions of Akt (43) as well as those of mechanistic target of rapamycin (44).

## Mitochondrial Dysfunction is Central to Pathogenesis

Warburg considered dysfunctional mitochondria (respiration) as the cause of cancer. Generation of superoxide radical (O^-^_2_) leading to ROS has become the symbol of mitochondrial dysfunction. The key factors that promote the ROS generation are high proton motive force (Δ*p*), a reduced coenzyme Q (CoQ), a high NADH/NAD ratio, and the presence of intra mitochondrial O_2_ (22). We have earlier presented a view that inhibition of autophagy has a crucial role in promoting cell cycle progression and metabolic reprograming (45). One of the consequences of inhibition of autophagy is also the inhibition of mitophagy (46). In addition, inhibition of FoxO3a by activated Akt down regulates the anti oxidant enzymes MnSOD and catalases (47); mutational deregulation of mitochondrial genome by ROS disables mitochondrial ability to produce ATP. ROS in fact, are suggested to act as rheostat in deciding the cell fate and metabolism in hematopoietic stem cells by regulating the Bcl-2 proteins (48). As already indicated, high energy (ATP/ADP) conditions promote activation of Akt (43) and the mTOR (44). The Akt-mTORC1 signaling also modulates the mitochondrial survival by regulating the activity of GSK3β, which has crucial regulatory role in activation of proapoptotic mitochondrial proteins (49, 50). Mitochondria are required for amino acid metabolism, citrate production, urea production, heme synthesis, and FeS assembly etc (51, 52). In cancer cells, GSK3β remains inhibited; inhibition of mitophagy during proliferation of cells may also lead to activation of aspartate, malate, and citrate shuttles, which are very much essential for carbon and nitrogen recycling during biosynthetic process. The life style healthy practices like aerobic exercise, yoga, which promote expenditure of energy, the dietary restriction, which limits the energy (calorie) intake and therapeutic drugs like metformin, PPARγ agonists, which promote activation of AMPK and autophagy, therefore, play crucial roles in amelioration of metabolic pathologies (53, 54).

## Conclusion

**Figure 1:**
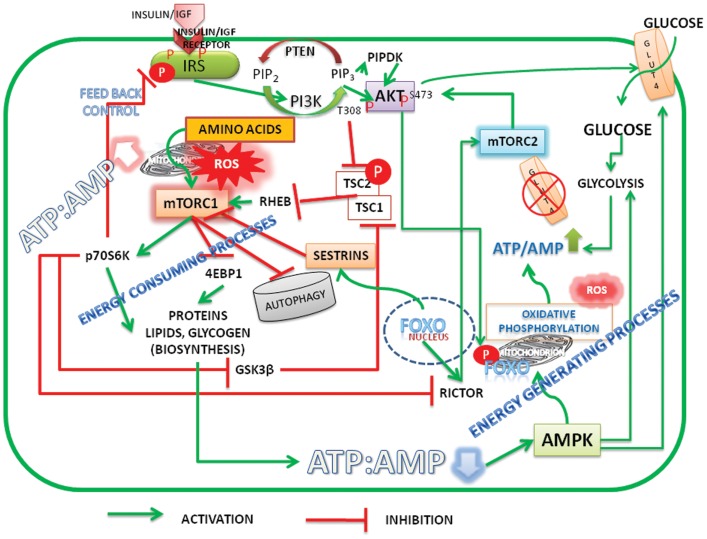
**Schematic representation of energy status of cells modulating cell signaling and mitochondrial function**. Under high energy (ATP/AMP ratio)/nutrient levels the insulin/insulin growth factor signaling (IIS) activates PI3K-Akt pathway. Initiation of PI3K-Akt signaling takes place when mTORC2 is active and phosphorylates Akt on Serine473. FoxO is the transcription factor of rictor, a critical component of mTORC2. Akt is further phosphorylated at Threonine 308 by IIS mediated PIPDK (originally PDK1). High ATP/AMP ratio stabilizes the phosphorylations and activated Akt phosphorylates FoxO, which leads to its exclusion from the nucleus. Akt inactivates tuberous sclerosis complex (TSC) 1/2 resulting in activation of mTORC1. mTORC1 promotes biosynthetic activity, inhibits autophagy. When ATP/AMP ratio is high, mitochondria stop synthesizing ATP and generate reactive oxygen species (ROS) and activate metabolite shuttles to replenish the amino acids and citrate, the precursors of protein and membrane lipids. Reduction in the ATP/ADP ratio, on the other hand, results in activation of AMPK, which activates autophagy. FoxO translocates into nucleus transcribes sestrins which inhibit mTORC1 and activates mTORC2 by transcribing rictor. The activation of glycolysis and glucose transport by Akt S473 and AMPK increases the ATP/ADP ratio there by reactivating the cycle. Under normal and healthy dietary conditions a perfect balance between ATP production, ROS generation, and biosynthetic processes is cyclically maintained by activation–inactivation cycles of autophagy modulated by alternate activation and inactivation cycle of AMPK and mTORC1. Under surplus nutrients/inflammatory conditions, a deregulated hyper activated mTORC1 leads to either carcinogenesis or insulin resistance. A growing tumor, with increased population of cells is heterogeneous with mixed population of cells, either deprived of oxygen or having access to it. It maintains a metabolic symbiosis with hypoxic cells surviving on glucose uptake and anaerobic glycolysis, while those having access to oxygen thrive on lactate accumulating in the neighborhood (microenvironment). Akt, protein kinase B (T308, S473 – Phosphorylated sites Threonine 308 and Serine 473); AMPK, AMP activated protein kinase; FoxO, fork head transcription factors of O group; GSK3β, glycogen synthase kinase3β; GLUT, glucose transporter; IGF, insulin growth factor; IRS, insulin receptor substrate; mTORC1, 2, mechanistic target of rapamycin Complex 1 and 2 (mTOR: formerly known as mammalian target of rapamycin); PIP2, phosphatidylinositol 4,5 bisphosphate; PIP3, phosphatidylinositol 3,4,5 trisphosphate; PIPDK, phosphoinositide dependent kinase 1 (the abbreviation PIPDK is preferred over the original PDK1 in the article to avoid confusion with the pyruvate dehydrogenase kinase, which is also abbreviated as PDK1 in the literature); PI3K, Phosphatidylinositol 3-kinases; Rictor, a Component of mTORC2; p70S6K, The p70 Ribosomal S6K; ROS, reaction oxygen species; Sestrins, stress response proteins.

In summary, it should be recognized that there is a unifying concept in the hypotheses of Pasteur, Warburg, and Crabtree that proliferative cells require oxygen, nitrogen source, and glucose (albeit lesser than that required for fermentation). Mitochondria, which are central to Warburg's theory of damaged respiration, have diverse functions. Taking cues from signal pathways, it is suggested that high ATP/ADP ratio activate both Akt and mTORC1, inhibit autophagy/mitophagy, up regulate ROS, and promote biosynthetic activity (see Figure 1). Mitochondrial metabolism shifts from ATP production to anaplerotic reactions that replenish fatty acids and non-essential amino acids (55) for biosynthesis of macromolecules and membrane structures of proliferating cells. During the discussion of the article, two new publications (56, 57), highlighted the role of nutrients/energy, Akt, and mTORC1 in modulating biosynthetic activity of cells.
